# Polycyclic Aromatic Hydrocarbons in Coastal Sediment of Klang Strait, Malaysia: Distribution Pattern, Risk Assessment and Sources

**DOI:** 10.1371/journal.pone.0094907

**Published:** 2014-04-18

**Authors:** Seyedeh Belin Tavakoly Sany, Rosli Hashim, Aishah Salleh, Majid Rezayi, Ali Mehdinia, Omid Safari

**Affiliations:** 1 Institute of Biological Sciences University of Malaya, Kuala Lumpur, Malaysia; 2 Food Science and Technology Research Institute, ACECR Mashhad Branch, Mashhad, Iran; 3 Chemistry Department, Faculty of Science, University Malaya, Kuala Lumpur, Malaysia; 4 Department of Marine Science, Marine Living Group, Iranian National Institute for Oceanography, Tehran, Iran; 5 Faculty of Natural Resources and Environment, Ferdowsi University of Mashhad, Mashhad, Iran; University of Illinois at Chicago, United States of America

## Abstract

Concentration, source, and ecological risk of polycyclic aromatic hydrocarbons (PAHs) were investigated in 22 stations from surface sediments in the areas of anthropogenic pollution in the Klang Strait (Malaysia). The total PAH level in the Klang Strait sediment was 994.02±918.1 µg/kg dw. The highest concentration was observed in stations near the coastline and mouth of the Klang River. These locations were dominated by high molecular weight PAHs. The results showed both pyrogenic and petrogenic sources are main sources of PAHs. Further analyses indicated that PAHs primarily originated from pyrogenic sources (coal combustion and vehicular emissions), with significant contribution from petroleum inputs. Regarding ecological risk estimation, only station 13 was moderately polluted, the rest of the stations suffered rare or slight adverse biological effects with PAH exposure in surface sediment, suggesting that PAHs are not considered as contaminants of concern in the Klang Strait.

## Introduction

Polycyclic aromatic hydrocarbon (PAH) contamination is a major hazard that is a concern for aquatic life in marine sediments, particularly in areas close to anthropogenic sources [1],[2]. Many PAHs are at the same time persistent, bioaccumulative, and toxic for humans and aquatic organism [3], [4], [5], [6], [7].

In environmental research, the aromatic fraction of C_11_–C_22_ was selected as being representative of aromatic hydrocarbon compounds for the purpose of assessment of ecological and human risks. This fraction is associated with the release of petroleum products to the environment, and is potentially toxic due to its mobility and stability in sediments [8], [9]. Physicochemical properties of these fractions are provided in the Supplementary data (Table S1).

Similar to other pollutants, the sources of PAHs are divided into major groups; anthropogenic and lithogenic. Anthropogenic sources of PAHs originated from pyrogenic and petrogenic sources. Pyrogenic PAHs are usually made up of high molecular weight PAHs with 4–6 rings that includes, fluoranthene (Fla), pyrene (Py), benzo(a)anthracene (BaA), chrysene (Chy), benzo(b)fluoranthene (BbF), benzo(k)fluoranthene (BkF), benzo(a)pyrene (BaP), dibenzo(a,h)anthracene (DibA), benzo(g,h,i)perylen (BghiP) and Indeno[1,2,3,(c,d)]pyrene; (InP) [2], [10]. The pyrogenic are mainly detected in incomplete combustion of organic compounds, such as fossil fuels (heating oil, cooking, coal burning, vehicle emissions, waste tire), and biomass burning (fireplace, controlled burning) [2], [11], [12]. Pyrogenic sources are thought to be more thermodynamically stable and toxic than petrogenic sources due to their high concentrations of non-alkylated PAHs [11], [13], [14].

Petrogenic PAHs involve naphthalene, acenaphthylene, acenaphthene, and fluorine, and belong to the alkyl-substituted PAHs or low molecular weight PAHs, possessing 2–3 rings. These hydrocarbons are thought to have originated from oil spills from fresh or used crankcase oil, crude and fuel oil, chronic or accidental leakages of marine and land pipelines, and domestic and industrial wastes [2], [10], [12], [14].

The Klang Strait is surrounded by the west coast of Malaysia and the Straits of Malacca in Southeast Asia. The strategic location of this strait has made it into one of the busiest shipping routes in the world, corresponding to huge economic demands from the Middle East and the Far East [15]. This strait also experienced the rapid development of industrialization, urbanization, and motorization over the past few decades. Thus, this area is under constant threat from multiple sources of energy, such as petroleum [16], [17]. In this research, a hypothesis is defined based on the serious threat posed by PAHs contamination to the Klang Strait.

Hence, this study tries to estimate the concentration and distribution of PAHs in coastal sediments from the Klang Strait to identify the possible sources of PAHs in the Port, and to conduct an ecological risk assessment to recognize the possible adverse ecological effects on the biological community due to the exposure to PAHs concentrated in Klang Strait sediments.

Ecological assessment of PAHs compounds in this study has been highlighted via two main problems: the first problem was related to the scarcity of background and updated databases on the PAHs concentrations in Klang Strait sediments. This problem is due to the limitations in collecting samples such as strong currents, high traffic density of shipping activity. The second problem was related to the lack of SQGs (Sediment Quality Guidelines) for coastal waters of Malaysia, since no SQGs were available to assess biological effects. Therefore, the results from this study will be applied in the form of the managerial tools in order to control the pollution occurrence and protect living organisms to assure the safe future of the marine environment of Peninsular Malaysia. Moreover it can be practical as background data for future studies.

## Materials and Methods

### Ethics Statement

The Ethical Review Committee of University Malaya approved this study. All necessary permits were obtained for the described field study from University Malaya and Port Klang Authorities (Permit Number UM.G/KB4/6/1). No specific permission was required for water and sediment surveys as they were conducted at these stations. Likewise, the field studies did not involve endangered or protected species, and no lethal sampling was conducted.

### Study area and sample collection

The Klang Strait is divided into three main Ports, most of which are well-sheltered by several mangrove islands and mudflats, forming natural enclosures. Experimental samples were collected from three sites (North Port, South Port and West Port) in the Klang Strait, which is located on the western coastal region (03° 00′N to 101° 24′E) of Peninsula Malaysia at the north end of the Malacca Strait, and over 573 km^2^ [18].

The sites were chosen based on in their unique activities. The North Port is a terminal of container to transship the coal, oil, and other chemical products, while the South Port is a terminal jetty for fishing boats, ferries, and yachts, and is linked to the Klang river, which is known as the most polluted river in Malaysia [15], [19].

The West Port is a main container terminal for massive cargo transport by ocean ships. Similarly, an industrial complex, including cement industries and a food, and palm oil factories have been constructed at the West Port [15].

**Figure 1:**
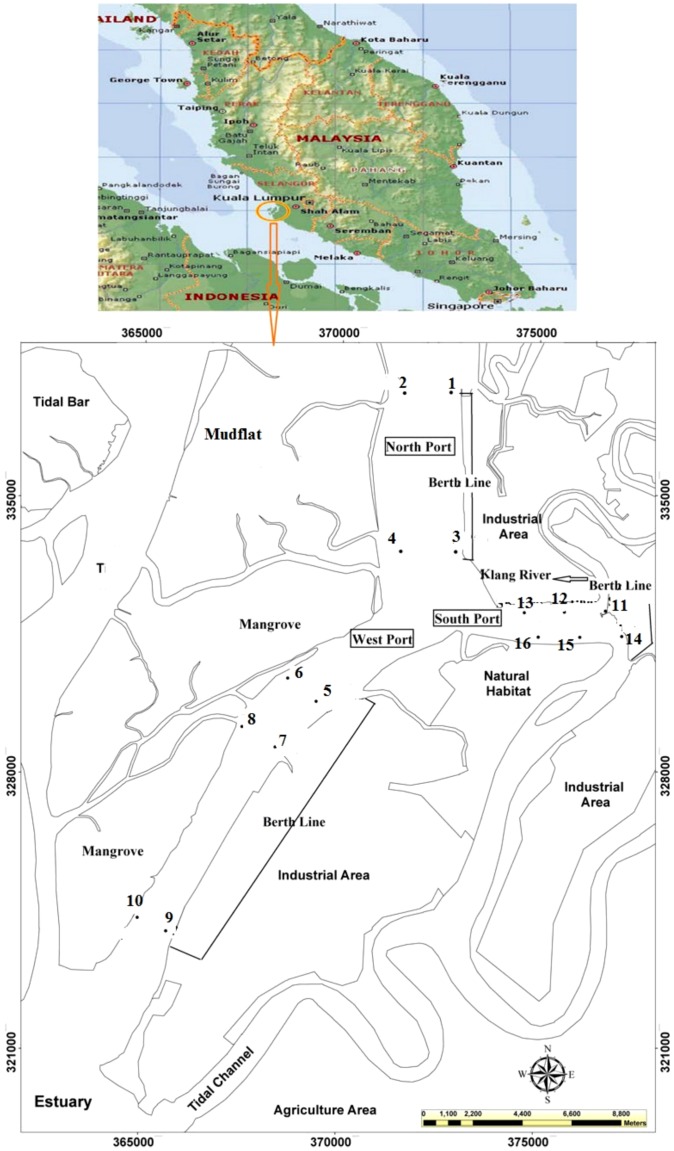
Location of the sampling stations in Klang Strait.

In this research, 22 stations were selected from three transects parallel to the coastline, with three different distances (Figure 1), and a station was selected as a control point 21 km from the Klang Strait at a remote location. The sampling stations were adjacent coastal industrial outlets and busy shipping lanes. The sampling stations were identified a progressive number: six up-gradient stations in North Port (Station 1–6), six stations in South Port close to Klang River (station 7–15), and nine down-gradient stations in West Port (station 16–21) (Table 1).

**Table 1:** Physicochemical description of sampling stations.

Sites	Station number	Latitude	Longitude	Silt and clay (<64 µm)%	TOC (%)	Depth (m)	Salinity ( 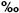 )	Description of stations
North Port	1	3° 3′1.49″N	101°21′18.70″E	58.20	41.79	14.3	30.15	Liquid and dry berth line
	2	3° 3′1.33″N	101°20′56.04″E	49.63	50.36	20.5	30.18	Remote
	3	3° 3′1.47″N	101°20′33.11″E	73.77	26.22	10.3	31.24	Mangrove
	4	3° 0′53.11″N	101°21′20.25″E	59.78	40.21	13.5	30.81	Container berths
	5	3° 0′52.64″N	101°20′58.04″E	50.89	49.10	21.6	31.02	Remote
	6	3° 0′52.49″N	101°20′34.54″E	65.19	34.80	11.2	31.36	Mangrove
West Port	7	2°58′44.00″N	101°19′21.02″E	53.57	46.42	12.5	30.86	Dry berth and Cement factory outlets
	8	2°58′54.12″N	101°19′9.06″E	45.96	54.03	19.5	30.98	Remote
	9	2°59′3.12″N	101°18′58.38″E	63.42	36.57	7.8	30.86	Mangrove
	10	2°58′6.34″N	101°18′48.14″E	56.33	43.66	13.3	30.44	Liquid berth and palm oil factory oulets
	11	2°58′14.90″N	101°18′34.56″E	41.10	58.89	20.3	30.58	Remote
	12	2°58′23.07″N	101°18′20.99″E	70.81	29.18	8.8	30.79	Mangrove
	13	2°55′34.43″N	101°17′18.76″E	52.31	47.68	15.5	30.51	Container berths
	14	2°55′39.38″N	101°17′7.57″E	50.69	49.30	21.1	30.63	Remote
	15	2°55′45.02″N	101°16′55.55″E	70.36	29.63	6.8	30.77	Mangrove
South Port	16	2°59′59.08″N	101°23′18.88″E	95.39	4.60	7.5	26.10	Dry berths, Klang river
	17	2°59′58.17″N	101°22′45.35″E	93.16	6.83	10.5	26.12	Klang river
	18	2°59′57.66″N	101°22′12.45″E	64.69	35.30	12.4	30.11	Semi-urban
	19	2°59′38.25″N	101°23′32.32″E	69.50	30.49	10.3	29.45	Liquid berth
	20	2°59′37.70″N	101°22′57.93″E	69.72	30.27	11.3	29.54	Mangrove
	21	2°59′37.23″N	101°22′23.78″E	57.73	42.26	10.4	30.50	Mangrove
Control Point	22	3° 6′55.95″N	101°12′44.70″E	51.60	48.39	17.5	31.54	Remote

The study area lies within the humid tropical part with rainy (North monsoon, November to March) and dry seasons (south monsoon, April to October). In the Klang Strait, the average salinity is 30.25‰ (±1.36), the average temperature is 30.04°C (±0.62), and the average surface dissolved oxygen (DO) is 5.38 mg/l (±0.17), while the monthly average surface and bottom pH values were between 7.85 to 8.25. The sediment samples were taken with a Peterson grab sampler (0.07 m^2^) at two dates per season during November 2011 until October 2012. The top 10 cm of the sediment sample was removed with a stainless steel spoon for subsequent analysis. All of the samples were packed into aluminum boxes and transported on ice to the laboratory. The samples were air-dried, crushed, and sieved (<2 mm) to remove residual roots and stones, and immediately stored at −20°C in pre-cleaned amber glass bottles until further treatments. For PAHs analysis, three replicates of grab samples were used to investigate the spatial and temporal distributions.

### Experimental method

#### Chemical and reagents

A standard solution of 16 USEPA priority PAHs (Table 2) and a five-surrogate standard (acenaphthene-d10, chrysene-d12, perylened12, naphthalene-d8 and phenanthrene-d10) were purchased from Ultra Scientific, Inc. (North Kingstown, RI, USA). A standard reference material (SRM) was obtained from the National Institute of Standards and Technology (NIST, Gaithersburg, MD, USA). Neat (99%) hexamethylbenzene was purchased from Aldrich Chemical Company (Milwaukee, WI, USA). All solvents (acetone, dichloromethane, methanol and hexane) applied in the analyses were of analytical grade and redistilled twice.

**Table 2:** Concentration of PAHs (µg/kg dw) in surface sediments of Klang Strait.

Station	Nap	Acy	Ace	Flr	Phn	Ant	Fla	Pyr	BaA	Chy	BbF	BkF	BaP	DibA	Bghip	Inp	∑PAHs
**1**	ND	ND	2.1	34.2	47.5	100.4	ND	482.8	613.0	ND	ND	ND	ND	ND	ND	ND	**1280.4**
**2**	1.3	ND	ND	9.7	13.3	12.7	ND	131.3	31.5	ND	ND	ND	ND	ND	ND	ND	**199**
**3**	1.7	ND	8.1	118.5	89.0	58.8	42.7	62.9	ND	ND	ND	ND	ND	ND	ND	ND	**381.7**
**4**	0.4	16.32	ND	1.1	10	59.8	12.5	12	66.7	50.0	852.8	ND	1495.8	190.2	ND	99.8	**2852.2**
**5**	0.8	ND	0.3	16.8	12.6	12	10	195.6	ND	ND	11.0	ND	ND	ND	ND	ND	**259.1**
**6**	4.0	ND	0.4	57.3	36.2	37.9	26.0	31.8	179.4	ND	ND	ND	ND	ND	ND	42.3	**415.2**
**7**	1.3	ND	ND	27.6	127.6	267.8	10.3	12.7	204.0	ND	ND	47.6	ND	ND	ND	ND	**698.9**
**8**	5.7	ND	1.0	39.0	6.8	71.4	13.5	15.8	50.5	ND	ND	ND	122.1	ND	64.2	77.4	**467.4**
**9**	1.2	ND	ND	7.7	5.1	8.2	ND	28.1	191.3	198.0	ND	ND	ND	67.8	ND	ND	**507.4**
**10**	0.8	ND	ND	23.7	5.0	10.1	ND	236.5	278.8	ND	ND	ND	ND	ND	ND	68.7	**623.7**
**11**	0.7	ND	0.7	48.8	15.6	37.8	17.6	25.1	29.5	ND	ND	367.6	ND	ND	ND	ND	**543.4**
**12**	2.0	ND	1.0	39.8	24.8	233.9	21.5	341.5	774.7	ND	ND	1.3	1.0	7.3	ND	ND	**1448.8**
**13**	16.1	645.3	14.8	520.8	225.6	308.0	166.5	243.8	390.9	ND	11.6	28.6	825.2	ND	49.4	ND	**3446.9**
**14**	3.0	ND	0.5	33.8	75.8	181.3	15.8	14.0	146.3	ND	11.3	ND	59.9	ND	ND	31.8	**573.5**
**15**	2.2	ND	ND	8.7	9.6	3.8	ND	ND	78.3	ND	12.8	ND	ND	130.2	ND	348.4	**594.0**
**16**	109.0	ND	1.2	120.6	66.3	100.5	92.2	99.7	974.8	116.0	ND	ND	716.8	ND	ND	ND	**2397.1**
**17**	9.6	ND	4.0	225.6	94.3	501.2	ND	226.2	1292.0	549.2	ND	ND	ND	ND	ND	ND	**2902.1**
**18**	1.3	ND	ND	20.8	8.5	11.6	ND	119.3	191.8	ND	378.6	ND	ND	ND	ND	ND	**731.9**
**19**	2.2	ND	ND	35.6	39.7	33.3	16.5	17.1	267.3	ND	10.2	ND	103.9	ND	ND	ND	**525.8**
**20**	2.1	ND	ND	37.1	38.6	30.2	16.3	17.0	226.8	ND	9.3	ND	89.9	ND	ND	ND	**467.5**
**21**	2.4	ND	0.6	37.2	16.7	16.5	16.0	11.2	194.6	ND	22.0	ND	ND	129.6	ND	ND	**483.5**
**22**	1.8	ND	ND	12.5	1.7	4.1	ND	ND	56.8	ND	23.4	ND	ND	0.0	ND	ND	**100.3**
**Mean**	7.7	30.04	1.6	67.13	43.6	95.5	21.2	105.7	282.7	41.5	61.0	20.2	155.8	23.9	5.2	32.0	**994.02**
**SD**	22.6	137.39	3.453	131.0	53.01	142.3	38.0	144.0	363.8	121.7	191.9	77.5	377.7	53.7	16.7	76.1	**918.1**
**Min**	ND	ND	ND	1.1	1.7	3.8	10	11.2	ND	ND	ND	ND	ND	ND	ND	ND	**100.3**
**Max**	109.0	645.3	14.8	520.8	225.6	501.2	169	599	1292.0	549.2	852.8	367.6	1495.8	190.2	64.2	348.4	3446.9
*TEL	34.6	5.87	6.71	21.2	86.7	46.9	113	153	74.8	108	320	280	88.8	6.22	430	*	1684
*PEL	391	128	88.9	144	544	245	1494	1398	693	846	1880	1620	763	135	1600	*	16770

TELs (threshold effects level) and PELs (probable effects level), ** <MDL, below the method detection limit; ND, not detect.

The alumina (120–200 mesh) and Silica gel (80–100 mesh) were extracted for a period 72 hours in a Soxhlet apparatus, activated in the oven at 150°C and 180°C for a period 12 hours, respectively, and then deactivated by distilled water at a ratio of 3% (m/m). The deionised water was obtained from a Milli-Q system.

#### Extraction and fractionation

For the extraction procedure, about 5 g of dried sediment samples were Soxhlet-extracted by dichloromethane (150 ml) for a period of 72 hours. A mixture of surrogate standard was added to all of the samples as a recovery surrogate standards. Elemental sulfur was removed from the extracts using activated copper granules. After extraction, the extract was concentrated up using a rotary vacuum evaporator. The volume of the extract was adjusted to 2–3 ml, and solvent-exchanged into 10 ml n-hexane, which caused it to further decrease to approximately 1–2 ml.

Clean up and fraction procedures were performed with a 1∶2 alumina/silica gel column. PAHs were obtained by eluting it with 60 ml of hexane/dichloromethane (1∶1). PAHs fraction were re-concentrated using a rotary evaporator, while the volume was concentrated to up to 1 ml. The fraction was further reduced to 0.2 ml under a stream of filtered purified nitrogen gas. An aliquot of 0.2 ml of each extract was applied to gas chromatography–mass spectrometer (GC–MS) analysis. Likewise, hexamethylbenzene was added as an internal standard prior to the GC–MS analysis.

#### Instrumental analysi

The PAHs were estimated using a Hewlett–Packard 5890 series gas chromatograph interfaced with 5972 mass-selective detector (MSD) in the selective ion-monitoring (SIM) mode. A fused silica capillary column (50 m, 0.32 mm, 0.17 µm) coated with HP-5MS (film thickness 0.25 lm) was used for separation, with helium as a carrier gas at a flow rate of 2 ml/min, a head pressure of 12.5 psi, and a linear velocity of 39.2 cm/s at 290°C.

The temperature was programmed to vary from 80 to 290°C at 4°C/min, and was held at the final temperature for 30 minutes. The injector temperature and transfer line temperature were 250°C and 180°C, respectively. The mass spectrometer operated at electron energy of 70 eV, with an ion source temperature of 250°C.

The interface temperature and injection were programmed at 290°C. The oven temperature was initially isothermal at 80°C for 5 minutes, programmed from 80 to 290°C at 3°C/min, and held at the final temperature (290°C) for 30 minutes. A 1 ml sample was manually injected in the split-less injector with a 1-minute solvent delay. The mass spectrometer was operated at electron impact (EI) of 70 eV, with an ion source temperature of 250°C. The ranges of mass scanning were between m/z 50 and m/z 500.

#### Quality assurance

For every set of samples, a procedural method blanks (solvent), sample duplicates, spiked matrices (standards spiked into solvent), and standard reference material (SRM1941) were used to assess quality control and assurances. Individual PAHs were quantified according to the retention time and m/z ratio of PAHs mixed standard (Sigma), while a standard calibration curve was used to calibrate the concentrations of each PAH.

The method detection limits (MDLs) for the investigated organic pollutants were determined according to USEPA [20]. The MDL of PAHs were estimated based on six analyses of the minimal solutions (**Σ**16PAH) of the standard PAH mixture, which was subjected to the full analytical procedure in the range of 0.0641 ng/g to 1.0183 ng/g. These values were within the acceptable range of the EPA method [20]. The percentage of relative standard deviation (RSD) for all of the investigated PAHs were lower than 20.0% in the fortification experiment and in the replicates used to determine the MDL. The reported results were corrected with the recoveries of the surrogate standards. The average recoveries of surrogate standards varied from 63.28–96.75. The recoveries of all the PAHs and RSD falls within the range of 78.4–95.2% and 2.6–13.5%, respectively, which met the acceptance criteria of the EPA method [20]. A detailed recovery and RSD values were provided in the Supplementary data (Table S1).

#### Measurement of TOC and sediment grain size

The sediment grain size was determined using a multi-wavelength particle size analyzer (model LS 13 320), and the results were divided into sand (>64 µm), silt (2 µm<size<64 µm), and clay (<2 µm) fractions for the determination of PAHs in the contaminated sediments [21].

Freeze-dried sediment samples were grounded, and was treated with 10% (v/v) HCl to remove carbonates from the sediment samples. After that, the samples were dried at 60°C in an oven. Total organic carbon (TOC) was determined in the surface sediments using a carbon analyzer (Horbia Model 8210).

### PAHs source identification

Ratio values such as an Ant/Ant+Phn and a Fla/Fla+Pyr had been used to distinguish between pyrogenic and petrogenic sources [22], [23]. Sediments with Ant/Ant+Phn ratio <0.100 were mainly contaminated by petrogenic inputs, while a ratio >0.100 indicates a dominance of combustion [10], [22], [23]. A Fla/Fla+Pyr ratio of 0.500 is usually defined as the petroleum/combustion transition point. This boundary appears to be less definitive than 0.100 for Ant/Ant+Phn.

The Fla/Fla+Pyr ratio is below 0.4 for most petroleum samples, between 0.400–0.500 for liquid fossil fuel (vehicle and crude oil) combustion, and exceeds 0.500 in kerosene, grass, most coals, and wood combustion [22], [23].

### Ecological risk assessment

A Screening Level Ecological Risk Assessment (SLERA) was performed to evaluate the possibility of adverse ecological effects according to the framework developed by the United States Environmental Protection Agency (USEPA) [11], [24], [25]. The average concentration of individual PAHs in Klang Strait was used as the exposure concentration in a conservative assumption.

The sediment quality guideline, which was previously developed, was applied in the risk characterization step. In the present study, a consensus approach based on MacDonald et al. was used according to the following specific values of TEL (threshold effects level) and PEL (probable effects level) [26], [27]. In the risk characterization step, the hazard quotient (HQ) was calculated based on the maximum concentration of each individual PAHs (MCPAHs) and the estimated consensus based sediment quality guideline (CCPEL) according to the following equation [11], [27]: 
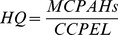
(1)


The PEL quotient (PELq) is a unique factor to describe the contamination effect of PAHs on biological organisms according to the analyses of chemical data and matching toxicity from 1,068 sediment samples from coastal and estuaries water in the USA [28]. The PELq factor is the average of the ratios between the PAHs concentration in the sediment sample and the related PEL value [11], [29]. PELq factors were divided into four categories, which can be used to describe the sediment as non-adverse effect (PELq<0.1), slightly adverse effect (0.1<PELq>0.5), moderately effect (0.5<PELq>1.5), and heavily effect (PELq>1.5) [11],[30]. This factor is practical for comparing different historical episodes or other study areas, and to facilitate the decision maker's work in sediment quality assessment [31].

The Nemerow composite index (Nemerow Index) for all of the sediments samples was estimated based on [32]. This method used the individual contamination index, which directly reflect the sediments' contamination degree. The equation is as follows: 
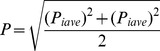
(2)


Where *P_i_*_ max_ is the maximum concentration of the individual contamination indices, while *P_i ave_* is the average concentration of the individual contamination indices. When *P_i_* = C*_i_*/S*_i_*, *P_i_* is the individual contamination index of PAHs contaminates *i*, C*_i_* is the determined concentration of PAHs from at least 5 sampling points, and S*_i_* is the required standard based on the evaluation criteria. In the present study, the adopted evaluation criteria were specific values of the sediment quality guideline, as Malaysian coastal waters have no stipulated guideline on PAHs in sediments. The Nemerow pollution index (P) is classified as the quality of sediment into 5 pollution grades safety domain (P<0.7), precaution domain (0.7<P≤1.0), slightly polluted domain (1.0<P≤2.0), moderately polluted domain (2.0<P≤3.0), and seriously polluted domain (P>3.0) [32], [33].

### Statistical analysis

Statistical analyses were performed using Microsoft Excel and SPSS 17 software (SPSS, Chicago, IL) for statistical data evaluation. Multivariate techniques are sensitive to the distribution of non-parametric geochemical data. Thus, evaluating normal distribution and data transformation are essential to appropriately pretreat data. The data were arranged based on the samples and estimated values, and converted into a single matrix formed by the data points (cases) and the concentration values of the 16 PAHs analyzed (variables), forming a [22×16] data matrix. The methods were selected based on the results of the Shapiro-Wilk Normality test, the levene test for homogeneity of variances, and the Bartlett's test of equal variances. Normally, the distributed data were evaluated via one-way ANOVA, with an alpha level of 0.05. Data that did not pass the tests of normality and homogeneity was evaluated with the Kruskal-Wallis one-way nonparametric ANOVA. The differences were determined to be significant, where p<0.05. The data transformation was also done using the Box–Cox method with an optimal transformation for data that were not normally distributed. This method was performed based on the Kannel et al. 2007 to reduce the mean squared error [71]. Statistical tests, multivariate analyses, diagnostic ratios, and risk calculations include non-detect (or left-censored) data with substitution by zero. The nonparametric correlation method (Kendall's tau-b) was used to obtain the correlation coefficient and the significance of the correlation among physicochemical parameters in the sediments. Multivariate techniques, such as cluster analysis (CA) and principal components analysis (PCA) were used to estimate structure and relationships in a multivariate data. PCA is also known as a dimensional reduction because this method is able to decrease the dimensionality of the primary set of data (measured PAH contents in sediment samples) and compress data into a lower dimensional matrix (principal components) [34]. Specifically, the KMO and Bartlett's test of sphericity were applied to extract all factors with eigenvalues that exceed 1, and were rotated by the Varimax method. CA analysis was used to classify a set of data into different groups based on similarity. The geo-statistical analysis was performed using Surfer 8 software (GPS value of stations) based on the geospatial methods to better understand the contaminant pathways and to provide a comprehensive contour map of the spatial distribution of contaminants over a large range.

## Results and Discussion

### Spatial distribution of PAHs in sediments

In the Klang Strait, fine-grained sediments are predominated in almost all stations, with the highest amount of fine sediment in the vicinity of stations near the mangrove edge and the mouth of the Klang River (Table 1). TOCs' distribution was synchronous with that of fine-grain-sized sediment in most parts of the study areas (with the notable exception of stations 9 and 12) (Table 1). The details related to the distribution and concentration of TOC in the Klang strait was recorded by Tavakoly Sany et al. in 2013 [35]. Generally, the PAH concentration showed an insignificant correlation with the percentage of TOC and fine-grained sediments (p<0.05, r = 0.15 and r = 0.11) in the surface sediments of the Klang Strait. Thus, it might be probable that fine-grained sediments and TOC are not effective parameters in controlling the distribution of PAHs in the Klang Strait.

**Figure 2:**
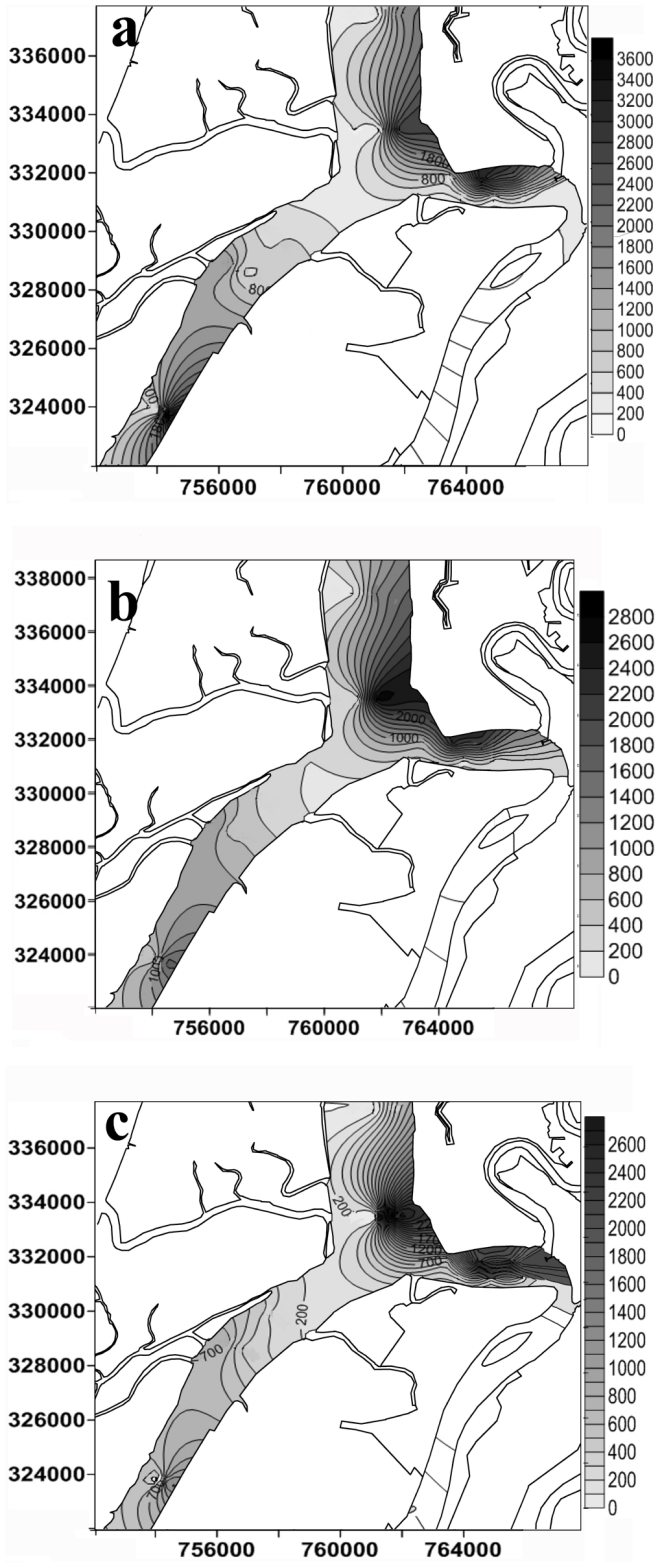
Spatial variations of PAHs compounds in surface sediment of the Klang Strait (a: Total PAHs, b: PAHs _combust_, c: PAHs _toxic_).

In the Klang Strait, the concentration of total PAHs ranged from 100.3 to 3446.9 µg/kg dw, with an average concentration of 994.02±918.1 µg/kg dw (Table 2). The highest concentration of PAHs was estimated at station 13, while the lowest concentration was observed at the control point. The PAH contamination was estimated for surface sediments all over the strait, with higher concentrations observed at the stations close to the berth line (except station 12), especially in front of the container terminal in the West Port (station 13: 3446.9 µg/kg dw), in front of the dry and liquid terminal in the North Port (station 1: 1280.4 µg/kg dw and station 4: 2851.2 µg/kg dw), and in stations located adjacent to the mouth of the Klang River in the South Port (16: 2397.1 µg/kg dw and 17: 2902.1 µg/kg dw). PAH concentrations were generally lower in more remote stations and mangrove side, except for station 12, which had an elevated concentration of PAHs (1448.8 µg/kg dw) (Figure 2A). A Kruskal–Wallis test showed that there are significant differences (*p*<0.05, df = 21, sig  = 0.003) among the concentrations of PAHs at all stations.

The concentrations of high-molecular-weight PAHs (HMWPAHs) are usually estimated in order to assess the combustion values [2], [10], [11]. In our study, combustion values ranged from 80.3 µg/kg dw to 2767.8 µg/kg dw, with an average concentration of 767.2 µg/kg dw (Table 3). High-molecular-weight PAHs represented between 27.66% and 97.08% of the total concentration of PAHs, with a mean value of 77.18%. Moreover, the combustible PAHs constituted a significant portion of the total PAHs (more than 50%) at all stations, with the exception of stations 13 (in front of the container terminal) and 7 (close to cement outlets) in the West Port, and stations 3 in the North Port (Figure 2b).

**Table 3:** Molecular indices of PAHs, content in surface sediments of Klang Strait.

Station	∑ PAH *_combust_*	∑ PAH *_toxic_*	Ant/Ant+Phn	Fla/Fla+Pyr	L/H-PAH
1	1195.8	613.0	0.68	0.00	0.15
2	162.8	31.5	0.49	0.00	0.23
3	105.6	0.0	0.40	0.40	2.61
4	2767.8	2755.3	0.85	0.51	0.03
5	206.6	11.0	0.49	0.05	0.20
6	279.4	221.7	0.51	0.45	0.49
7	274.6	251.6	0.68	0.45	1.55
8	343.5	250.0	0.91	0.46	0.36
9	485.2	457.1	0.61	0.00	0.05
10	584.1	347.6	0.67	0.00	0.07
11	439.8	397.1	0.71	0.41	0.24
12	1046.8	784.3	0.90	0.08	0.29
13	1715.9	1256.3	0.58	0.41	1.07
14	279.2	249.3	0.71	0.53	1.05
15	569.7	569.7	0.28	0.00	0.04
16	2099.5	1907.6	0.60	0.48	0.19
17	2267.4	2041.3	0.86	0.00	0.41
18	689.7	570.4	0.58	0.00	0.06
19	415.0	381.5	0.46	0.49	0.27
20	359.4	326.1	0.44	0.49	0.30
21	410.1	382.8	0.50	0.59	0.18
22	80.3	80.3	0.71	0.00	0.25
Mean	767.2	631.1	0.70	0.16	0.33
Standard deviation	233.2	213.8	0.22	0.28	0.32
Minimum	80.3	11	0.28	0.4	0.04
Maximum	2767.8	2755.3	1	1	2.61

**∑ PAH** ***_combust_***: Combustion value of PAHs calculated as sum of concentration of high-molecular-weight PAHs include Fla, Pyr, BaA, Chy, BbF, BkF, BaP, InP, DibA, BghiP; **∑ PAH** ***_toxic_***: Toxic value of PAHs calculated as sum of concentration of BaA, Chy, BbF, BkF, BaP, InP and DibA; L/H: ratio of low molecular weight hydrocarbon (**n**-C10–**n**-C14) to high molecular weight hydrocarbon (**n**-C16–**n**-C22).

Some high-molecular-weight PAHs, such as BaA, Chy, BbF, BkF, BaP, InP and DibA are regarded as known as toxic PAHs, due to their mutagenic and carcinogenic effects on humans and other organisms [11], [36]. Their concentration made up an average of 61.25% of the total concentration of PAHs, ranging from 0–97.3%. These concentrations were highest in stations 1, 4, 13, 15, 16, and 17, whereas at certain remote stations (2, 3, 5 and 22), the toxics' PAH concentrations were significantly lower.

Distribution maps were used to better visualize of the distribution of contamination at the spatial scales (Figure 2C). At the South Port, the distributions of PAH compound (*PAHs _combust_* and *PAH _toxic_*) sediments exhibited a homogeneous pattern of decreasing concentration in the north-to-south direction, and concentrations were high at the stations16 and 17, which are parallel to the mouth of the Klang River (Figure 2 and Table 3). This pattern supports the view that the Klang River may be the primary source of contamination in the Klang Strait, and influences the concentration and distribution of PAHs as water and suspended solids were easily exchanged between the South Port and the polluted Klang River, which contains industrial effluents and untreated municipal waste. Water currents in the vicinity of the South Port are weak; therefore, there is enough time for the deposit of organic components by suspended solids to enter surface sediments. Organic compounds are not easily deposited onto bottom sediments where there are strong water currents [15].

At both the West Port and North Port, the spatial distribution of PAHs exhibited a decreasing east–west gradient. These stations were located close to the near shore area, and are thus strongly influenced by port activities, especially at stations 4, 13 and 14, which were close to the terminal containers in the North and West Port. Additionally, land-based run-offs directly release organic compounds in the vicinity of these stations. Further research revealed significant differences in the sources and concentration of PAHs in sediment samples of near-shore vs. offshore areas. PAH concentrations in sediment samples collected from near-shore sites (city hinterland) were greater than the PAH concentrations in offshore areas [11], [30], [37], [38], [39]. This implies that PAH concentrations in near-shore areas are influenced by lateral transport, such as run-off and the transportation of water due to daily rainfall [12], [40]. In 2005, Ikaneka indicated that heavy rainfall and floods significantly contributed to the pollution of marine sediments. Moreover, the results of their work indicated that near-shore areas received both burnt material (pyrogenic) and oil products (petrogenic), whereas offshore stations were primarily influenced by the burnt materials [9], [24], [40].

Overall, the distinct pattern in the distributions of measured parameters revealed that multiple sources contributed significantly to the PAH loads in the Klang Strait. These sources include the large-scale inflow from industries, such as the palm oil, cement, and food manufacturers that are located along the coastline of the North and West Ports, vessel-based discharges, Klang River outflow, land-based run-off, sedimentation, and siltation.

### Source identification

Several methods were used to identify the source of PAHs according to the correlation analysis, diagnostic PAHs ratios, PCA analysis, and significant differences in the concentration of the individual PAHs.

A correlation analysis (Table S2) revealed a significant correlation between some individual PAHs related to pyrogenic and petrogenic compounds. This implied that the sediment samples are polluted by anthropogenic sources of PAHs because unpolluted sediment samples do not exhibit an oil fingerprint, and only some PAH compounds may be found in this sediment [12], [40].

Mahyar et al. in 2010 studied the characteristics and possible origins of PAHs in the developed and developing areas around the coastal waters of Peninsular Malaysia [12], [40]. The collected sediment core samples were used to assess the historical profile of PAHs from 1875 to 2007. Their research agrees well with our results, as because they revealed that older sediment samples (1875–1899) did not exhibit an oil fingerprint, and that only some PAH compounds were found. Sediment samples that are polluted by oil products generally exhibit high concentrations of phenanthrene and its methylated derivative. Thus, there is a high correlation between crude oil and petrogenic and pyrogenic compounds, whereas this correlation in old sediment samples with natural inputs (with no oil fingerprints) ranges from low to negative values [12], [40].

Ratios of PAHs, such as Ant/Ant+Phn and Fla/(Fla+Pyr) had been applied to provide an accurate estimation of PAH sources (Table 3) [41]. The ratio of Ant/Ant+Phn was greater than 0.1 for all stations, implying that the origin of the PAHs in the sediments of the Klang Strait is primarily combustion. However, the ratio of Fla/(Fla+Pyr) showed the mixed pattern of pyrogenic and petrogenic sources in most stations. In the present study, ratio analyses showed the contradictions in certain stations.

In several researches, PAH ratios have been considered for “ideal” samples dominated by a single source, such as smoke, vehicle emission, and wood. However, some authors indicated that the limits between the diagnostic PAHs ratios are imprecise, as most environmental samples contain PAHs from mixed sources, none of which dominates the PAH profile [2], [40], [41]. Likewise, the validity of many diagnostic PAHs ratios has been examined for source-distinction based on the source material in tropical Asian waters [12], [14], [42].

Although these ratios are usable for the differentiation of pyrogenic and petrogenic sources, they are not definitive, since several exceptions have been found. For example, Saha et al. in 2009 reported that, although all of the crude oil samples have Ant/(Ant+Phn)<0.1 and Phn/Ant>10, implying a petrogenic source, one crude oil sample (Miri) has Anh/(Anh+Phn)>0.1 and Phn/Ant<10, showing a pyrogenic source, and one wood sample has Anth/(Anh+Phn)<0.1; implying a petrogenic sources of PAHs [12], [14], [41]. Hence, these PAHs ratios are not authoritative enough to be exact; they only provide a rough idea for sources-distinction. These contradictions are most likely due to inconsistencies associated with the conventional ratios, large range of thermodynamic stability (discrimination ability) of different parent PAHs, and different homolog distributions between pyrogenic and petrogenic sources [2], [14], [42]. Despite these exceptions, the diagnostic PAHs ratios are now routinely analyzed. Several researchers suggest using more indices and cross-plot of diagnostic PAHs ratios to provide a more robust interpretation [10], [41], [43].

**Figure 3:**
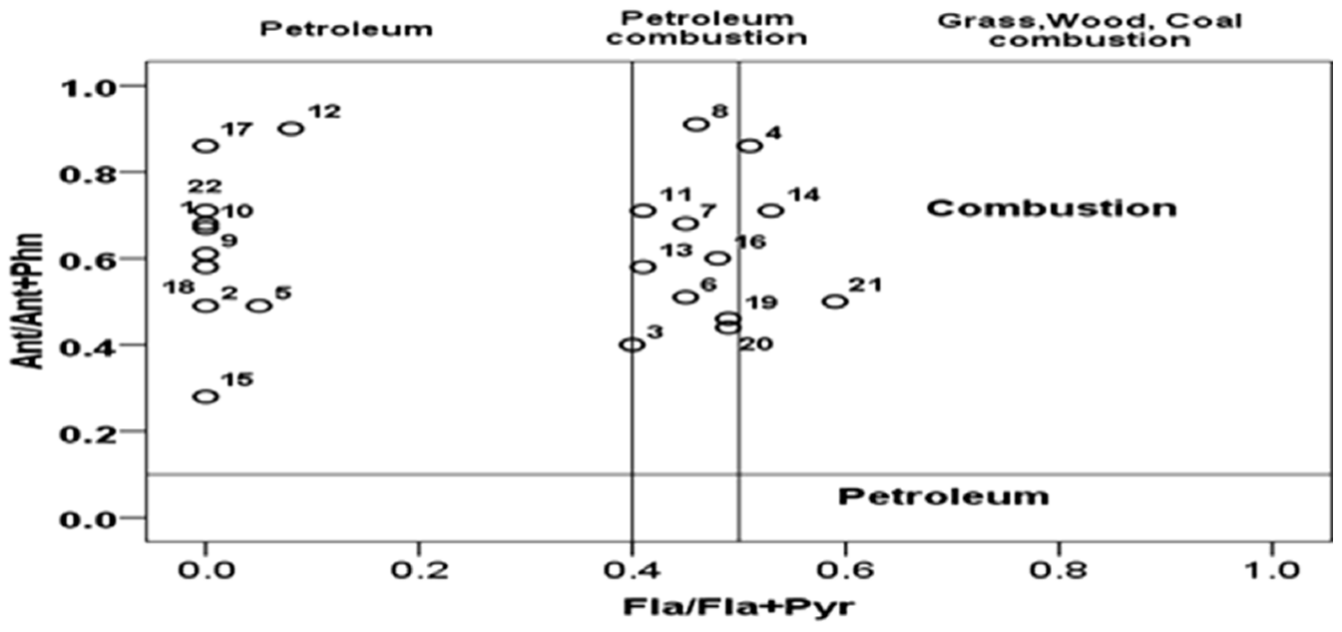
Plots of PAH isomer pair ratios for source identification (Ant/(Ant+Phy) vs Flu/(Flu+Pyr).

In the present study, the PAH ratio of Fla/(Fla+Pyr) was plotted against Ant/Ant+Phn for all of the sampling stations in Klang Strait (Figure 3). Figure 3 showed a mixed pattern of contamination from pyrogenic and petrogenic sources (petroleum combust) at some of the stations (3, 6, 7, 8, 11, 13, 16, 19 and 20). Simultaneously, wood combustion fingerprints (more frequent PAHs representing wood, coal and grass combustion) were strongly present in stations 4, 14, and 21. Other stations (1, 2, 5, 9, 10, 12, 15, 17, 18 and 22) have primarily originated from combustion sources with a significant contribution of petroleum inputs. Furthermore, An Ant/(Ant+Phn) ratio >0.10 almost always corresponds to a Fla/(Fla+Pyr) ratio >0.40, supporting the conclusion that combustion products are a main component of the contamination [10], [14], [41].

The purpose of principal component analysis (PCA) is to represent the total variation of the PAHs data with a minimum number of the factor loadings. By critically quantifying the percentage of contribution of individual PAH sources and identifying the occurrence of the sources responsible, each factor can be realized [2], [23], [44]. As shown in Table 4, PCA analysis classified the dataset of PAHs into five principal components (PCs) that control 81.89% of the variability in the data.

**Table 4:** Rotated component matrix of 16 PAHs from the Klang Strait sediment.

PAHs	PC1	PC2	PC3	PC4	PC5
Nap	.095	0.000	0.138	**0.954**	−0.034
Acy	**.957**	0.037	0.009	0.006	0.012
Ace	**.918**	0.061	.185	−0.013	0.031
Flr	**.910**	−0.042	0.332	0.114	0.069
Phn	**.824**	−0.106	0.322	0.091	.124
Ant	.429	0.063	**.814**	−0.064	.429
Fla	**.866**	0.017	.466	0.465	0.07
Pyr	.201	−0.335	**.779**	−0.021	−0.236
BaA	.058	−0.142	**.861**	0.392	−0.112
Chy	−0.087	0.094	**.865**	0.029	0.043
BbF	−0.092	**0.871**	−0.016	−0.016	0.036
BkF	.012	0.521	−0.139	0.108	**0.852**
BaP	0.288	**0.777**	.026	.395	0.013
DibA	−0.152	**0.847**	−0.096	−0.137	−0.217
BghiP	0.185	0.584	−0.185	−0.058	0.173
InP	0.081	0.609	0.139	−0.221	−0.492
Variance (%)	26.73	21.08	17.25	9.66	7.17

*Rotation method: Varimax with Kaiser normalization.

*Bold loadings >0.70.

The first factors (PC1) explained a total variance of 26.73% in the data. These factors were strongly weighted the context of Acy, Ace, Flr, Phn and Fla, and were believed to be from petrogenic sources of PAHs, and were also been identified as tracers for volatilization or spill of petroleum products [2], [12], [14], [23], [41], [42]. Phn and Fla are the thermodynamically stable triaromatic isomer, and their high concentration are regarded as characteristic of petrogenic sources [14], [23]. As indicated by a previous researcher, Phn and Fla were more abundant in crude oil, crankcase oil, and unburnt fuel, and originated from soot collected from diesel engines [12], [41], [45].

The second factor (PC2) responsible for 21.08% of the total variance was strongly related to BbF, BaP, DibA, Bghip, with moderate loading of InP and BkF. This factor are predominately composed of high molecular weight PAHs with 4–6 rings, and are basically known to be derived from the incomplete combustion and pyrolysis of fuel [44], [46]. The source of this individual PAHs have been found in both vehicular emission sources (diesel and gas engine), road dust, and gasoline combusted ships [2], [44]. InP and BkF have been found in gasoline vehicle soot, and both gas and diesel engine emissions [2], [12], [47], [48]. Bghip and BaP have been identified as a tracer of auto emissions because there were determined to be rich in road dust from urban-areas and traffic tunnels. The abundance of these compounds was considered as an index for the determination of autoexhaust contribution of gasoline-engines. Therefore, this factor is selected to represent the combustion source of PAHs [2], [12], [14], [41], [48].

The third factor is responsible for 17.25% of the total variance, and is predominately composed of Ant, Pyr, Chy, and BaA (4-ring PAHs), with moderate loadings of Fla. More researchers have used Ant as the marker of wood combustion source [2] (Harrison et al., 1996). Also, several literature have identified Pyr, BaA, Chy, Fla (4-rings) and Bap as a tracer of auto emissions, as it was found to be enriched in road dust collected from Kuala Lumpur, Bangkok, Shanghai [2], [12], [14], [47], [48], and traffic tunnels [10], [44], [47], [48]. The PAH profiles of road dust in these cities showed that the ratio of PAHs (4 rings) to PAHs (5–6 rings) exceeds 1 [12], [47]. In the present paper, we showed that the ratio of PAHs (4 rings) to PAHs (5–6 rings) in the surface sediment, 0–126.44 with a mean of 7.805 is significantly greater than that in the road dust from Kuala Lumpur (1.58). This result indicates that there might be an additional PAH source (not traffic-related), leading to a higher ratio of these PAHs [2]. Several researchers have identified Pyr, BaA, Chy, and Fla as markers of coal combustion because these individual PAHs are dominant in coal-combustion profiles [2], [12], [14], [47], [48]. In Malaysia, coal is the main energy source, and is used widely for industrial and domestic and industrial purposes, such as cement, palm oil, tire, and power industry [2], [12], [14]. It is reasonable to assign these PAHs to coal combustion and traffic-related source of PAHs.

PC4 explained 9.66% of the total variance, with a high correlation with Nap. Nap is used as a tracer of oil spills, such as Acy and Ace. It can also originate from termite activities on vascular land plants and woody material in tropical areas and the Amazonian region [49], [50]. The fifth factor (PC5) is responsible for 7.17% of the total variance and was strongly related to BkF, which are known to be derived from the incomplete combustion and pyrolysis of fuel [44], [46].

The PCA analysis described 45.5% of the total variance (PC2, PC3 and PC5) in the PAH load, including individual PAHs that are known to originate from coal combustion and vehicular emissions or traffic-related sources.

Similarly, 36.39% of the total variance (PC1 and PC4) in PAHs load related to petrogenic sources and the variance in PAHs was unrelated to unknown and biogenic sources.

There are two origins for vehicular sources in the Klang Strait, one is the traffic-related PAHs in road dust entering the surface sediment by land-based runoff, others due to the exhaust emission from cargo vessels, ships, ferries, and fishing boats [2], [12], [14], [47]. Furthermore, the cargo vessels play the main role as PAHs contributor in the Klang Strait compared to traffic related PAHs, as cargo vessels are responsible in transferring most coal to coal–fired power plants located along the West coastal water of Malaysia, such as Connaught bridge power, Kapar and Tanjung Bin, Manjung, and Jimah. Most fishing boats and ferries are still unequipped with catalytic converters [51], [52].

Coal and wood combustions mainly originated from agricultural, industrial, and habitats activities. Recently, coal usage near the Klang Strait is concentrated in the Kapar Power Plant in the Klang Valley region with high consumption of coal (more than16 million tons). Additionally, It is also the only power plant in Malaysia with triple fuel firing capability (natural gas, oil, and coal). It started operating in in 1987 and was the first coal–fired power plant in Malaysia [51], [52]. Moreover, Mahyar et al. in 2010 indicated that, in the near-shore and offshore areas near Klang, high levels of PAHs originated from pyrogenic sources, such as the combustion of fossil fuel and the burning of biomass and wood. The dominance of pyrogenic sources in this area is due to the products of combusted petroleum from industries and automobiles emissions [12], [40].

Petrogenic sources include petroleum products and crude oil, which are identified as major fossil fuels being used in Malaysia (around 49% of total energy consumption) [14]. Zakaria et al. in 2002 reported that several sources lead to the input of petrogenic PAHs into Malaysian coastal water, namely spillage of unburnt fuel and waste crankcase oil, leakage of crankcase oils from vehicles, with subsequent land-based runoff, wastewater of industry outlets, and vessel maintenance [12], [14], [47].

In addition, other studies have reported that contamination from various seas contribute to increase petrogenic pollution in the coastal waters of western Malaysia [16], [53]. For example, in 1999, Abdullah revealed that land-based run-off and contamination from an offshore oil field near Sumatra Island contributed to the petroleum contamination in the Malacca Strait, which is a narrow channel of marine water located between Sumatra Island, Indonesia, and Peninsular Malaysia. This strait is vulnerable to contamination caused by tanker operation and oil spills [16], [53], [54]. The Strait of Malacca also plays an important role in transporting pollution into the Klang Strait. Pauzi Zakaria in 2001 used a biomarker compound to identify the source of tar-balls off the coast of Malaysia.

They identified several petrogenic sources of contamination in the western coastal waters of Malaysia, such as accidental oil spills and tanker-derived sources in the Strait of Malacca and crude oils originating from land-based run-off from human activities. Additionally, they clearly showed that the western coastal waters of Malaysia have received approximately 30% of their petroleum pollution from Middle East crude oil (MECO) and South-East Asian crude oil (SEACO), which was probably transported to Malaysia via marine currents, tankers and shipping discharges, including ballast water and tank-washing water [16].

Generally, the results of the PCA analyses are in concordance with the evidence from the pair isomer ratio of PAHs, which revealed a mixture of pyrogenic-and petrogenic-derived PAHs. PAHs contamination in the sediment in the Klang Strait are derived primarily from vehicular sources, coal and wood combustions, and crude oil and coal (petroleum combustion sources), which can originate from land-based run-off, oil spill, tanker operation, shipping activities, and industrial discharges such as effluents from cement, food and oil factories.

### Ecological risk assessment

**Figure 4:**
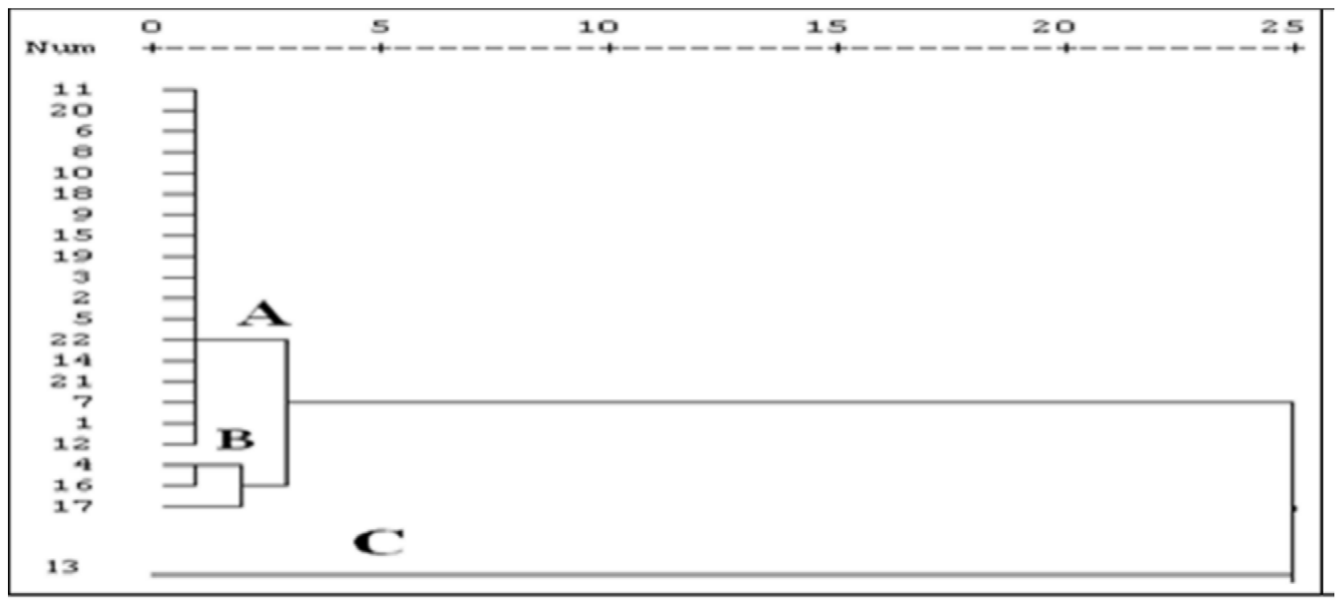
Cluster analyses to classify stations based on adverse biological effect of PAHs in surface sediment.

The results obtained from the ecological risk assessment of PAHs in the surface sediments of Klang Strait are summarized in Table 5. These results were arranged based on the concentration of PAHs measured at 22 stations. Generally, the risk assessment revealed that total PAHs are likely to cause slightly adverse effects on the biological communities at stations 4, 7, 12, 16, and 17 as these stations showed a value of 0.1<PELq<0.5 and 1.0<P≤2.0. Additionally, moderately adverse biological effects were shown for station 13 (0.5<PELq<1.5, 2.0<P≤3.0), but the rest of the stations showed rare, adverse ecological effects due to the PAH exposure in surface sediments (PELq<0.1, P<3). The cluster analysis allows for a better understanding of the similarity between sampling stations and PAHs contamination (Figure 4). The dendrograms clearly classified the sampling stations into three main clusters based on adverse biological effects. Station 13 (cluster C) was completely separated from the other stations as it demonstrated the moderate adverse biological effect by PAHs compound, while stations 4, 16, and 17 are in the same group (cluster B), with slightly adverse effects. The other stations are grouped into cluster A, and exhibited non-adverse effects (PELq<0.1, P<3), with the exception of stations 7 and 12. However, the average toxicity effects of PAHs in stations 7 and 12 are in the slight range, but these stations were arranged in cluster A, because their values are closer to 0.1 compared to the stations in cluster B. Based on a comparison with the sediment quality guidelines, only certain individual PAHs, such as Acy, Flr, Ant, BaA, BaP and DibA were associated with adverse biological effects, and only at stations 4 (close to the container terminal in the North Port), 7 (cement outlet), 13 (container terminal in the West Port), 12 (around the mangrove forest in the West Port) 16, and 17 (vicinity of the mouth of the Klang River) (Table 5).

**Table 5:** Ecological risk calculated for individual PAHs in the surface sediment of different station.

Stations	Nap	Acy	Ace	Flr	Phn	Ant	Fla	Pyr	BeA	Chy	BbF	BkF	BaP	DibA	BghiP	PELq	P
1	0.00	0.00	0.02	0.23	0.08	0.36	0.00	0.41	0.8	0.00	0.00	0.00	0.00	0.00	0.00	0.09	0.92
2	0.00	0.00	0.00	0.07	0.02	0.05	0.00	0.09	0.05	0.00	0.00	0.00	0.00	0.00	0.00	0.02	0,77
3	0.00	0.00	0.06	0.82	0.16	0.24	0.03	0.04	0.00	0.00	0.00	0.00	0.00	0.00	0.00	0.09	0,83
4	0.00	0.00	0.00	0.01	0.02	0.24	0.01	0.01	0.10	0.06	0.45	0.00	**1.96**	**1.41**	0.00	**0.28**	**1,56**
5	0.00	0.00	0.00	0.12	0.02	0.05	0.01	0.14	0.00	0.00	0.01	0.00	0.00	0.00	0.00	0.02	0,79
6	0.01	0.00	0.00	0.40	0.07	0.15	0.02	0.02	0.26	0.00	0.00	0.00	0.00	0.00	0.00	0.06	0,83
7	0.00	0.00	0.00	0.19	0.23	**1.09**	0.01	0.01	0.29	0.00	0.00	0.03	0.00	0.00	0.00	**0.12**	**1.02**
8	0.01	0.00	0.01	0.27	0.01	0.29	0.01	0.01	0.07	0.00	0.00	0.00	0.16	0.00	0.04	0.06	0,85
9	0.00	0.00	0.00	0.05	0.01	0.03	0.00	0.02	0.28	0.23	0.00	0.00	0.00	0.50	0.00	0.08	0,86
10	0.00	0.00	0.00	0.16	0.01	0.04	0.00	0.17	0.40	0.00	0.00	0.00	0.00	0.00	0.00	0.05	0,90
11	0.00	0.00	0.01	0.34	0.03	0.15	0.01	0.02	0.04	0.00	0.00	0.23	0.00	0.00	0.00	0.06	0,87
12	0.01	0.00	0.01	0.28	0.05	0.95	0.01	0.24	**1.12**	0.00	0.00	0.00	0.00	0.05	0.00	**0.18**	**1,14**
13	0.04	**7.26**	0.12	**4.31**	0.41	**1.26**	0.11	0.17	0.56	0.00	0.01	0.02	**1.08**	0.00	0.03	**1.03**	**2.03**
14	0.01	0.00	0.00	0.23	0.14	0.74	0.01	0.01	0.21	0.00	0.01	0.00	0.08	0.00	0.00	0.10	0,88
15	0.01	0.00	0.00	0.06	0.02	0.02	0.00	0.00	0.11	0.00	0.01	0.00	0.00	0.96	0.00	0.08	0,89
16	0.28	0.00	0.01	0.84	0.12	0.41	0.06	0.07	**1.41**	0.14	0.00	0.00	**1.07**	0.00	0.00	**0.30**	**1,42**
17	0.02	0.00	0.03	**1.57**	0.17	**2.45**	0.00	0.16	**2.15**	0.65	0.00	0.00	0.00	0.00	0.00	**0.48**	**1,57**
18	0.00	0.00	0.00	0.14	0.02	0.05	0.00	0.09	0.28	0.00	0.20	0.00	0.00	0.00	0.00	0.05	0,93
19	0.01	0.00	0.00	0.25	0.07	0.14	0.01	0.01	0.39	0.00	0.01	0.00	0.14	0.00	0.00	0.07	0,87
20	0.01	0.00	0.00	0.26	0.07	0.12	0.01	0.01	0.33	0.00	0.00	0.00	0.12	0.00	0.00	0.06	0,85
21	0.01	0.00	0.00	0.25	0.03	0.07	0.01	0.01	0.26	0.00	0.01	0.00	0.00	0.96	0.00	0.09	0.86
22	0.00	0.00	0.00	0.09	0.00	0.02	0.00	0.00	0.08	0.00	0.01	0.00	0.00	0.00	0.00	0.01	0,74
Mean	0.02	0.33	0.01	0.50	0.08	0.41	0.01	0.08	0.42	0.05	0.03	0.01	0.21	0.18	0.00	0.16	1,01

*Nemerow pollution index (P).

*The PEL quotient (PELq).

Moreover, the first three components of PCA analysis are strongly related to these individual PAHs that controls 63.36% of changes in PAHs contamination in surface sediment of the Klang Strait. According to the cluster and PCA classification, stations 4, 7, 13, 16 and 17 can be regarded as the main entrances for loading individual PAHs, especially Acy, Flr, Ant, BaA, BaP and DibA.

The present study shows that generally, the sediment is slightly polluted with PAHs (PELq = 0.16 and P = 1.01) in the Klang Strait. According to the results of ecological risk, it can be concluded that PAHs are not a primary pollution concern in the Klang Strait, and that only station 13 can be considered as a vulnerable station in the Klang area.

In this research, our hypothesis was defined based on the serious threat posed by petroleum contamination to the Klang Strait due to several evidences, which was reported in previous literature [16], [40], [53], [55].

The highest value for PAH contamination was estimated to be 1700 ng/g d.w. in near-shore Klang sediment samples from 1976–1999, with a predominant input of burnt materials and combusted petroleum discharges from industries, oil spill events and ship ballasting or bilge pumping. From 1976–1999, rapid development of the West Port and maritime-related industries that caused greater numbers of ocean going ships to enter the strait from the South China Sea and to the Far East to transport goods. Similarly, in 1997, a collision between two tankers in the straits of Singapore caused 25000 tons of heavy fuels oil to be released into the marine environment, of which 700 tons of oil were released into the Malacca strait, negatively affecting the coastal area of the Klang Strait [12], [40].

According to the present study, the hypothesis was rejected due to the fact that in most of the stations, the average PAHs concentration was significantly lower than the threshold effects level (TELs and PELs), and rarely reached a level likely to cause adverse biological effects.

Several studies recorded a significant decline in PAH discharges around the west coastal water of Malaysia after 2000 due to the establishment of an integrated management programme, weathering, and meteorological conditions [16], [40], [53], [55].

An integrated management programme was established in 1996, where several responsible organizations contributed to solving the environmental problems in Malaysia's marine environments. These organizations ratified specific regulations related to marine pollution to control petroleum and chemical contaminations in Malaysian coastal waters. These regulations focused on strategies that were based on international agreements to prevent and control pollution from ships, platform draining, and industrial inputs [16].

The weathering process can greatly deplete the concentration of PAH in a marine environment. The L/H-PAH ratio is accepted as a practical method for assessing weathering based on the differences between the low and high molecular weights of PAH compounds [16], [40], [53], [55]. These results show that the L/H-PAH ratio was below 1 at most of the stations, and exceeds 1 only at stations 3, 7, 13, and 14 (Table 3). Generally, a low ratio of L/H-PAHs is attributed to the high resistances of the high-molecular-weight PAHs to microbial degradation, which is consistent with other studies. Such a low ratio could also be due to the high solubility and volatility of the low-molecular-weight PAHs, which would lead to their depletion [16], [53], [56].

In addition, there is also evidence of a greater depletion of low-molecular-weight PAHs by the weathering process, which is clearly indicated by Pauzi Zakaria (2001) [16]. He revealed that the tar-ball samples from western Malaysian coastal waters had undergone significant weathering, as their L/H-PAH ratios were much lower (0.23–1.48) than the ratios of crude oil samples (8–44 for SEACO crude oil and 12.13–20.3 for MECO crude oil) [16]. In the present study, this ratio ranged from 0.02–2.61, which was significantly lower than that of the crude oil in this region. Several possibilities have been described to explain why the PAH compositions had a lower ratio of L/H in the western coastal waters of Malaysia, such as wandering due to long-haul transportation, ballast water, and tank washings [16], [53], [56].

Additional research has indicated that meteorological conditions play a major role in controlling PAH concentrations over spatial and temporal scales, and significantly negative linear relationships were found between wind speeds, temperatures, and PAH concentrations, because atmospheric turbulence causes a dilution of contaminant concentrations and hastens the weathering process, especially if the wind speed is greater than 5.8 km h^−1^ and the temperature is exceeds than 20°C [30], [57]. Thus, the higher temperature (approximately 30°C) of the Klang Strait as a tropical area could potentially increase the depletion of PAHs.

**Figure 5:**
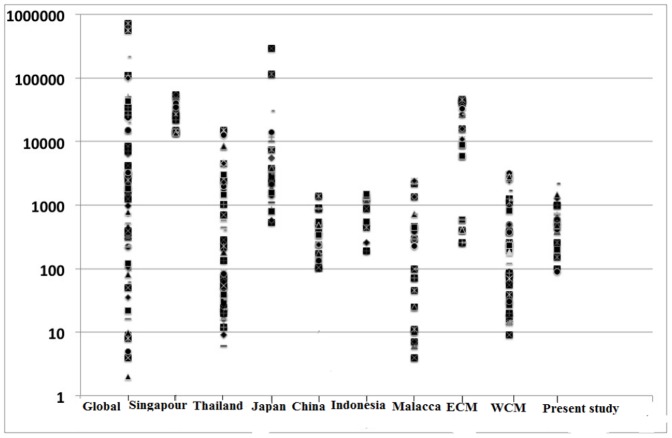
Total PAHs concentration (ng/g dw) in Klang Strait sediments in comparison to those of the reported concentrations for global, Southeast Asian countries, and Malaysian costal zones. Data for global sediments and others areas are derived from several literatures [12], [14], [40], [44], [45], [52], [55], [56], [58], [59].

Figure 5 shows the concentration of PAH in comparison with global, Southeast Asian countries, and Malaysian costal zones. Most values of PAHs concentrations in the present study, west coastal water of Malaysia, Malacca, Indonesia, China and Thailand ranged from tens to thousands of ng/g. Thus, PAHs values in Klang Strait sediments are comparable to these areas. On the other hand, the current PAHs concentration are 1–2 orders of magnitude lower than those in Japan, Singapore, and the eastern coastal water of Malaysia, as the value of PAHs in these countries ranged over thousands of ng/g. Thus, PAHs concentrations in Klang Strait can be categorized as low to moderate on a global scale.

## Conclusion

Exploratory source identification of PAHs in Klang Strait sediment was based on the distribution pattern of the individual PAHs, principal component analysis, and paired isomer ratios. The results of the distribution pattern showed that the pyrogenic sources are the major source of PAHs in most stations. Petrogenic input appears be a main source of PAHs in station 13 (in front of the container terminal) and stations 7 (close to cement outlets) in the West Port, and station 3 in the North Port because the LMWPAHs constituted a significant portion of the total PAHs in these stations.

The results of diagnostic ratios showed that PAHs are derived from a mixture of sources (both petrogenic and pyrogenic), with high frequencies of pyrogenic sources in most stations. The PCA analysis method showed that the contributions of spill of oil products, traffic-related pollution, and coal and wood combustion are dominant in Klang Strait sediments.

The pyrogenic sources (traffic-related pollution and coal combustion) are responsible for 45.5% of the total variance in the Klang Strait sediment sediments. As well as pyrolytic input as a main source, petrogenic input was also regarded as a source for PAHs in the Klang Strait due to direct petrogenic sources from oil leaks (oil spill, urban run-off, tanker operation and shipping activities) in agreement with their location.

Despite the inherent limitations of this research, the adopted approach in the present study highlighted that no adverse biological effects are associated with the exposure to PAHs levels in the Klang Strait, and only the areas around terminal container (station 13) are moderately polluted. Several factors caused the decline of these contaminants, such as an integrated management programme and regulation, meteorological conditions, and weathering. This paper represents a baseline study for future studies, which may include the whole Malaysian estuary and coastal waters for the purpose of promoting our knowledge of the distribution of PAHs in coastal and estuary ecosystems.

## Supporting Information

Table S1 **Physicochemical description of individuals PAHs.**
(DOCX)

Table S2 **Kendall's correlation coefficient between individual PAHs compounds.**
(DOCX)
