# Correction: Polycyclic Aromatic Hydrocarbons in Coastal Sediment of Klang Strait, Malaysia: Distribution Pattern, Risk Assessment and Sources

**DOI:** 10.1371/journal.pone.0105925

**Published:** 2014-08-14

**Authors:** The PLOS ONE Staff

There are errors in the Funding section. The correct funding information is as follows: This work and survey components were supported by the High Impact Research Grant (UM.C/625/1/HIR/162) from the Ministry of Higher Education (Malaysia) and University Malaya Research Grant (RP004A-SUS). Studies design and data collections were done by the academic staff at the Institute of Biological Science, University Malaya, Kuala Lumpur, Malaysia. Analyses undertaken and the decision to prepare and publish this manuscript were made by Institute staff in conjunction with the Chemistry Department of University Malaya.

Figures 1 and 5 are incorrect. The authors have provided corrected versions here.

**Figure 1:**
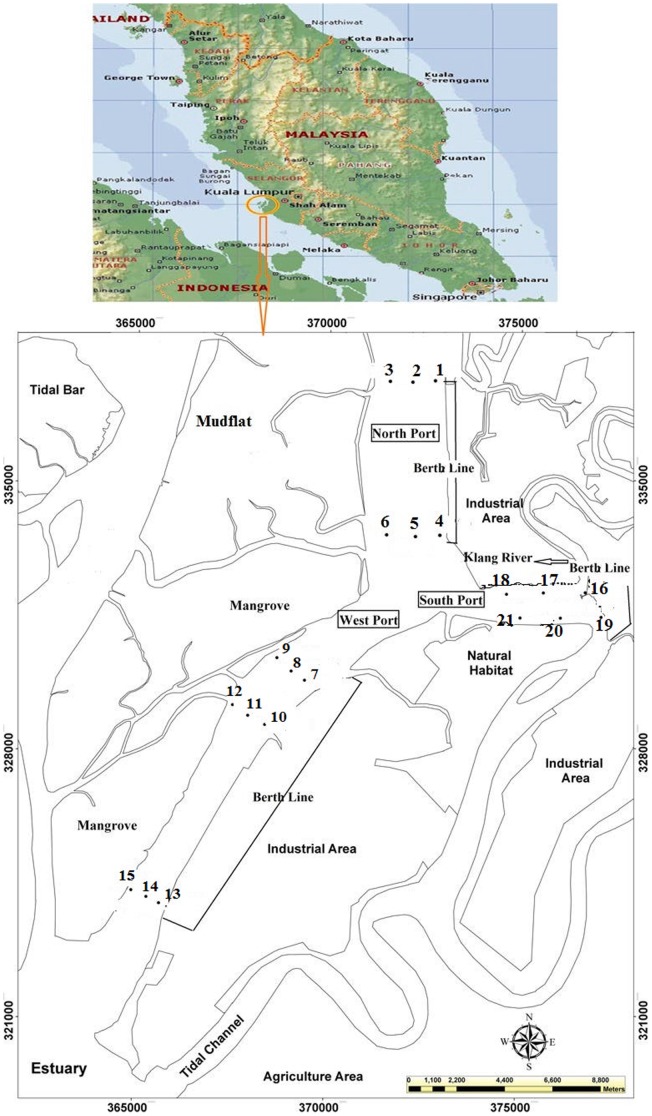
Location of the sampling stations in Klang Strait.

**Figure 5:**
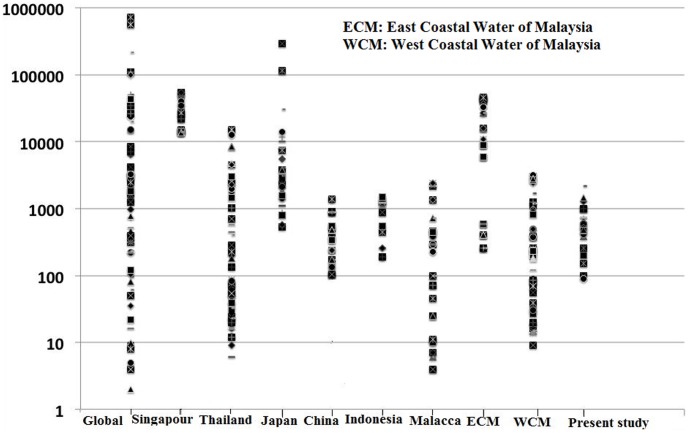
Total PAHs concentration (ng/g dw) in Klang Strait sediments in comparison to those of the reported concentrations for global, Southeast Asian countries, and Malaysian costal zones. Data for global sediments and others areas are derived from several literatures [12], [14], [40], [44], [45], [52], [55], [56], [58], [59].

## References

[1] Tavakoly Sany SB, Hashim R, Salleh A, Rezayi M, Mehdinia A, et al (2014) Polycyclic Aromatic Hydrocarbons in Coastal Sediment of Klang Strait, Malaysia: Distribution Pattern, Risk Assessment and Sources. PLoS ONE 9(4): e94907 doi:10.1371/journal.pone.0094907 24747349 10.1371/journal.pone.0094907 PMC3991632

