# Supplementary material for: Polycyclic Aromatic Hydrocarbons in Coastal Sediment of Klang Strait, Malaysia: Distribution Pattern, Risk Assessment and Sources
Source: PLoS One. 2014 Apr 18;9(4):e94907. doi: 10.1371/journal.pone.0094907 (PMC3991632; doi:10.1371/journal.pone.0094907)
Supplement: Table S1 — Physicochemical description of individuals PAHs. (DOCX) [file pone.0094907.s001.docx]

| PAHs and Abbreviations | Formula | ^A^Solubility mg/L  (25 ^0^C) | ^B^Log K_ow_ | ^C^Log K_oc_ | ^D^MDL ng/g | ^D^Recovery (±RSD)% |
| --- | --- | --- | --- | --- | --- | --- |
| Naphthalene (Nap) | C_10_H_8_ | 31 | 3.37 | 3.16 | 0.095 | 78(±7) |
| Acenaphthylene (Acy) | C_12_H_8_ | -- | -- | - | 1.018 | 83(±5) |
| Acenaphthene (Ace) | C_12_H_8_ | 3.93 | 3.92 | 3.67 | 0.0641 | 79(±7) |
| Fluorene (Flr) | C_13_H_10_ | 1.9 | 3.97 | 3.72 | 0.280 | 81(±3) |
| Phenanthrene (Phn) | C_14_H_10_ | 1.0-1.3 | 4.36 | 4.09 | 0.0894 | 95(±3) |
| Anthracene (Ant) | C_14_H_10_ | 0.05-0.07 | 4.44 | 4.16 | 0.174 | 91(±6) |
| Fluoranthene (Fla) | C_16_H_10_ | 0.26 | 5.22 | 4.89 | 0.064 | 84(±6) |
| Pyrene (Pyr) | C_16_H_10_ | 0.14 | 5.18 | 4.86 | 0.229 | 87(±5) |
| Benzo(a)anthracene (BaA) | C_18_H_22_ | 0.01 | 5.91 | 5.54 | 0.114 | 94(±2) |
| Chrysene (Chy) | C_18_H_22_ | 0.002 | 5.79 | 5.43 | 0.203 | 93(±4) |
| Benzo(b)fluoranthene (BbF) | C_20_H_10_ | 0.014 | 4.06 | 3.80 | 0.292 | 80(±5) |
| Benzo(k)fluoranthene (BkF) | C_20_H_10_ | --- | 6.4 | 6.41 | 0.145 | 95(±3) |
| Benzo(a)pyrene (BaP) | C_20_H_12_ | 0.0038 | 6.04 | 5.66 | 0.169 | 89(±2) |
| Dibenzo(a,h)anthracene (DibA) | C_22_H_14_ | 5 10^-4^ | 6.75 | 6.33 | 0.1864 | 92(±4) |
| Benzo(g,h,i)perylen (BghiP) | C_22_H_12_ | 2.6 | 6.29 | 5.90 | 0.247 | 78(±5) |
| Indeno[1,2,3,(c,d)]pyrene; (InP) | C_22_H_12_ | 5.3 | 6.5 | 6.09 | 0.241 | 88(±3) |

**Table S1,** Physicochemical description of individuals PAHs

**^A^**Solubility and Log K_ow_ from TPHCWG, 1997 [[8](#_ENREF_1)]

**^B^** K_ow_ is an expression of the relative affinity of a chemical for dissolution in the octanol and water phases of a 50/50 octanol/water mixture. It is measured as the ratio of the concentration of the chemical in the octanol phase to its concentration in the aqueous phase after equilibration.

**^C^** The Log organic carbon-to-water partitioning coefficient (Log Koc) is related to Log Kow for each hydrocarbon by the following equation: Log Koc = 0.00028 + 0.938Log Kow [[8](#_ENREF_2)].

**^D^** These factors were estimated in this research.

# 
