# Supplementary material for: Polycyclic Aromatic Hydrocarbons in Coastal Sediment of Klang Strait, Malaysia: Distribution Pattern, Risk Assessment and Sources
Source: PLoS One. 2014 Apr 18;9(4):e94907. doi: 10.1371/journal.pone.0094907 (PMC3991632; doi:10.1371/journal.pone.0094907)
Supplement: Table S2 — Kendall's correlation coefficient between individual PAHs compounds. (DOCX) [file pone.0094907.s002.docx]

Table S2. Kendall’s correlation coefficient between individual PAHs compounds

|  | Nap | Acy | Ace | Flr | Phn | Fla | Ant | Pyr | BaA | Chy | BbF | BkF | BaP | DibA | BghiP | InP | PAHs |
| --- | --- | --- | --- | --- | --- | --- | --- | --- | --- | --- | --- | --- | --- | --- | --- | --- | --- |
| Nap | 1.00 |  |  |  |  |  |  |  |  |  |  |  |  |  |  |  |  |
| Acy | .082 | 1.00 |  |  |  |  |  |  |  |  |  |  |  |  |  |  |  |
| Ace | .099 | .**847** | 1.00 |  |  |  |  |  |  |  |  |  |  |  |  |  |  |
| Flr | .249 | **.895** | **.922** | 1.00 |  |  |  |  |  |  |  |  |  |  |  |  |  |
| Phn | .209 | **.759** | **.817** | **.852** | 1.00 |  |  |  |  |  |  |  |  |  |  |  |  |
| Fla | **.519** | **.846** | **.802** | **.851** | **.759** | 1.00 |  |  |  |  |  |  |  |  |  |  |  |
| Ant | .111 | .371 | .482 | **.621** | **.605** | .321 | 1.00 |  |  |  |  |  |  |  |  |  |  |
| Pyr | .015 | .236 | .309 | .292 | .204 | .134 | **.765** | 1.00 |  |  |  |  |  |  |  |  |  |
| BaA | **.508** | .071 | .189 | .384 | .319 | .215 | **.701** | **.547** | 1.00 |  |  |  |  |  |  |  |  |
| Chy | .188 | -.075 | .099 | .267 | .150 | -.086 | **.618** | .335 | **.691** | 1.00 |  |  |  |  |  |  |  |
| BbF | -.098 | -.057 | -.136 | -.158 | -.196 | -.100 | -.127 | -.152 | -.170 | -.026 | 1.00 |  |  |  |  |  |  |
| BkF | -.070 | .024 | -.004 | .023 | -.015 | .033 | -.032 | -.138 | -.168 | -.091 | -.084 | 1.00 |  |  |  |  |  |
| BaP | .372 | .400 | .286 | .340 | .264 | **.512** | .115 | -.062 | .081 | .017 | **.708** | -.074 | 1.00 |  |  |  |  |
| DibA | -.125 | -.098 | -.187 | -.219 | -.285 | -.155 | -.232 | -.318 | -.213 | .008 | **.608** | -.119 | **.675** | 1.00 |  |  |  |
| BghiP | .035 | .386 | .399 | .414 | .347 | **.589** | .198 | .023 | -.081 | -.108 | .393 | .232 | **.534** | -.141 | 1.00 |  |  |
| InP | -.097 | -.088 | -.167 | -.183 | -.236 | -.172 | -.208 | -.237 | -.220 | -.114 | .157 | -.107 | .094 | **.650** | **.556** | 1.00 |  |
| PAHs | 0.05 | **0.50** | 0.25 | 0.24 | **0.69** | **0.50** | 0.05 | 0.18 | **0.52** | **0.54** | 0.04 | 0.35 | **0.58** | **0.54** | 0.19 | 0.00 | 1.00 |

# 
